# Rasch analysis of Stamps's Index of Work Satisfaction in nursing population

**DOI:** 10.1002/nop2.61

**Published:** 2016-07-27

**Authors:** Nora Ahmad, Nelson Ositadimma Oranye, Alyona Danilov

**Affiliations:** ^1^ Department of NursingBrandon UniversityBrandonManitobaCanada; ^2^ Department of Occupational TherapyUniversity of ManitobaWinnipegManitobaCanada; ^3^ Neil John Maclean Health Sciences LibraryUniversity of ManitobaWinnipegManitobaCanada

**Keywords:** nurses, nursing, Rasch analsyis, job satisfaction, Stamp's Index

## Abstract

**Aim:**

One of the most commonly used tools for measuring job satisfaction in nursing is the Stamps Index of Work Satisfaction. Several studies have reported on the reliability of the Stamps' tool based on traditional statistical model. The aim of this study was to apply the Rasch model to examine the adequacy of Stamps's Index of Work Satisfaction for measuring nurses' job satisfaction cross‐culturally and to determine the validity and reliability of the instrument using the Rasch criteria.

**Design:**

A secondary data analysis was conducted on a sample of 556 registered nurses from two countries.

**Methods:**

The RUMM 2030 software was used to analyse the psychometric properties of the Index of Work Satisfaction.

**Results:**

The persons mean location of ‐0.018 approximated the items mean of 0.00, suggesting a good alignment of the measure and the traits being measured. However, at the items level, some items were misfiting to the Rasch model.

## 1 Background

Job satisfaction remains an important topic in organizational studies and has been extensively studied in many fields, including nursing. Studies on job satisfaction dates back to as early as 1920 (Snarr & Krochalk, 1996) and have been studied with numerous tools and in different populations. The existing evidence shows that job satisfaction is influenced by multiple factors operating at the level of the job, individual, professional, organizational and the general work environment (Pittman, 2007; Ravari, Bazargan, Vanaki, & Mirzaei, 2012). Some of the specific factors that have been found to affect nurses' job satisfaction are job stress (Flanagan & Flanagan, 2002), management style of nursing leadership (Pietersen, 2005; Yamashita, Takase, Wakabayshi, Kuroda, & Owatari, 2009), empowerment (Cicolini, Comparcini, & Simonetti, 2014; Manojlovich & Laschinger, 2002), nursing autonomy (Castaneda & Scanlan, 2014; Hayes, Bonner, & Pryor, 2010), co‐worker interactions, group cohesion and salary (Curtis & Glacken, 2014; Wielenga, Smit, & Unk, 2008). The multiplicity of factors that impinge on nurses' job satisfaction have made the development of measurement tools that are valid and reliable across different work and cultural environments very challenging. However, several measurement tools have emerged over time, most of which have demonstrated high reliability and validity.

There are several reasons why job satisfaction among nurses has remained a persistent and hot topic in the nursing literature. Many researchers recognize the need to monitor job satisfaction of nurses because nurses' dissatisfaction could be disruptive to patient care delivery and reduce healthcare organizational effectiveness (Cheung & Ching, 2014; Curtis, 2007; Djukic, Kovner, Budin, & Norman, 2010; Taunton et al., 2004). Also, job satisfaction has been linked to different outcomes for the nurses, which includes nurses' perceived ability to express caring behaviours with patients (Amendolair, 2012), new immigrant nurses' acculturation (Ea, Griffin, L'Eplattenier, & Fitzpatrick, 2008) and ‘lower levels of job‐stress, burnout and career abandonment among nurses' (Foley, Lee, Wilson, Cureton, & Canham, 2004, p. 94). Nurses job satisfaction has also been associated with positive patient outcomes, such as reduced patient falls (Alvarez & Fitzpatrick, 2007). However, it is important that measurement tools used for job satisfaction are constantly reviewed to ensure that they are measuring what they are intended to measure and that users are made aware of any pitfalls, should they choose to use such tools.

### 1.1 Measurement tools for job satisfaction

A large body of research on job satisfaction has been accumulated, either using or attempting to validate well‐known measurement tools or new tools that assess nurses' job satisfaction. Our search of the literature on nurses' job satisfaction from 1986 to May, 2015 identified 100 studies that reported measurement of job satisfaction in nursing. Among these studies, there were 20 different instruments used to measure nurses' job satisfaction. Some of the tools that showed good reliability and validity and which were most commonly used include: Minnesota Satisfaction Questionnaire developed by Weiss and colleagues in 1967 (Kaplan, Boshoff, & Kellerman, 1991; Lamarche & Tullai‐McGuinness, 2009; Stamps, 1997; Weiss, Dawis, & England, 1967); Index of Work Satisfaction (IWS) developed by Stamps and Piedmont in 1970s (Slavitt, Stamps, Piedmont, & Haase, 1978; Stamps & Piedmonte, 1986); Quinn and Staines's Facet‐free Job Satisfaction Scale developed by Quinn and Staines in 1979 (Djukic et al., 2010; Kovner, Brewer, Wu, Cheng, & Suzuki, 2006); Mueller and McCloskey's Satisfaction Scale (MMSS) developed by Mueller and McCloskey in 1990 (Misener, Haddock, Gleaton, & Abuajamieh, 1996; Mueller & McCloskey, 1990; Price, 2002; Tourangeau, Hall, Doran, & Petch, 2006).

The Minnesota Satisfaction Questionnaire has Cronbach α range of 0.83–0.84 and validity between 0.32‐0.75 (Lamarche & Tullai‐McGuinness, 2009). Zurmehly (2008) noted that Hoyt reliability coefficient between 0.59–0.97 has been reported for the Minnesota Satisfaction Questionnaire, while Kaplan et al. (1991) reported a Cronbach α ranging between 0.82–0.90 for the different components, which demonstrate adequate reliability. The internal consistency of the MMSS was reported as 0.89 in Mueller and McCloskey (1990) and 0.90 in Misener et al. (1996). The test–retest reliability for the subscales ranged from 0.08–0.64 (Misener et al., 1996; Mueller & McCloskey, 1990). With regard to Quinn and Staines's Facet‐free Job Satisfaction Scale, Kovner et al. (2006) reported reliability coefficients for the scales ranging from 0.70–0.95. The psychometric properties of IWS have been reported in multiple studies (Ahmad & Oranye, 2010; Huber et al., 2000; Stamps, 1997; Wade et al., 2008), which reported on the internal consistency reliability and the validity of the IWS scales. Zangaro and Soeken (2005) explored the reliability and validity of the IWS through a meta‐analysis of 14 studies that used the IWS to measure nursing job satisfaction. The meta‐analysis by Zangaro and Soeken (2005) included only articles that reported the reliability of part B of the IWS and concluded that the part B of the IWS was reliable and valid in different settings, including university, community and acute care hospitals and for multisite studies. The internal consistency reliability and validity of the IWS scale and its subscales ranged from 0.50–0.92 Cronbach's α (Bjork, Samdal, Hansen, Torstad, & Hamilton, 2007; Hayes, Douglas, & Bonner, 2015; Itzhaki, Ea, Ehrenfeld, & Fitzpatrick, 2013; Manojlovich & Laschinger, 2007; Penz, Stewart, D'Arcy, & Morgan, 2008; Zangaro & Soeken, 2005). The highest subscale coefficient of 0.92 was reported by Manojlovich and Laschinger (2007), while the lowest Cronbach's alpha was reported by Medley and Larochelle (1995). The Cronbach's α originally reported by Stamps (1997) ranged from 0.82–0.91. Content validity (Kovner, Hendrickson, Knickman, & Finkler, 1994) and construct validity through factor analysis (Stamps, 1997) have been established.

Among these job satisfaction measurement tools, the IWS has been one of the most widely used. The IWS measures ‘the extent to which people like their jobs' (Stamps, 1997, p. 13) and provides a quantitative estimation of nurses' job satisfaction. The tool was amplified in 1986 by Stamps and Piedmont based on a critical review of occupational theories in the social sciences (Amendolair, 2012; Kovner et al., 1994; Slavitt et al., 1978; Stamps & Piedmonte, 1986). The strong theoretical foundation of Stamps's IWS was intended to address the seemingly atheoretical plunge of many of the extant job satisfaction measurement tools. Stamps and Piedmonte (1986, p. 19), noted that they ‘…proceeded to develop a valid and reliable scale for measuring nurses' work satisfaction, one general enough to be used in many settings…' The IWS scale assesses the level of nurses' professional satisfaction in six work dimensions: payment, professional status, task requirements, interactions, organizational policies and autonomy (Stamps & Piedmonte, 1986) and is rated on a seven‐point Litert scale. The level of professional satisfaction for each of the six dimensions (subscales' scores) and the overall professional satisfaction level (entire IWS score) have been reported in previous studies.

Hitherto, the statistical methods typically used for psychometric measurement in nursing research were based on the traditional statistical model. The Rasch analysis model provides an alternative to the traditional psychometric measurement that is sophisticated, comprehensive and is based on the Item Response Theory (Belvedere & de Morton, 2010; Hagquist, Bruce, & Gustavsson, 2009). The Rasch model was originally developed for measuring the psychometric properties of educational testing tools (Andrich, 2005), but nowadays, has been increasingly used in health sciences and many other disciplines. However, not many studies have been undertaken using the Rasch model in health sciences (Hagquist et al., 2009). A successful implementation of the Rasch measurement requires that the assumptions of local independence and unidimensionality are satisfied (Brentari & Golia, 2008). In addition to the criteria of unidimensionality and local independence, Rasch uses the criteria of differential item functioning (DIF), person separation index (PSI) and fit statistics to determine the reliability and validity of a measurement tool.

Few studies have been undertaken using the Rasch model in the health sciences (Hagquist et al., 2009) and very few studies have used Rasch to measure nurses' job satisfaction. There were three articles that applied the Rasch model in nursing (Clinton, Dumit, & El‐Jardali, 2015; Flannery, Resnick, Galik, Lipscomb, & McPhaul, 2012; Hagquist et al., 2009); however, despite the wide use of Stamps' IWS in nursing research and in diverse environments, no study has applied the Rasch model to evaluate its reliability and validity. The purpose of this study was to apply the Rasch model to examine the adequacy of Stamps's Index of Work Satisfaction for measuring nurses' job satisfaction cross‐culturally and to determine the validity and reliability of IWS using the Rasch criteria.

## 2 Methodology

This is a secondary data analysis that uses data from Ahmad and Oranye (2010) survey of registered nurses in two teaching hospitals in Malaysia and England. A total of 556 registered nurses participated in that study and are included in this analysis. The survey used four previously developed scales of Structural Empowerment scale, The Psychological Empowerment scale, Meyer and Allen Organizational Commitment Scale and the Index of Work Satisfaction scale. Details of the study design and description of the tools are reported in Ahmad and Oranye (2010).

### 2.1 Ethics

The study complies with the international human research ethics guideline and the Declaration of Helsinki code of ethics. The Ethical approval for the study was obtained from the University of Sheffield Ethics Committee, the NHS and Hospital directors in the two hospitals in England and Malaysia (Ahmad & Oranye, 2010).

### 2.2 Procedure

The current descriptive study uses the data related to part B of Stamps (1997) IWS tool to determine the adequacy of the IWS tool in measuring job satisfaction cross‐culturally, by applying the Rasch model. The IWS contains 44 items with six components of pay, autonomy, task requirements, professional status, interaction and organizational policies. There are six items in the pay subscale, eight in autonomy, six in task requirements, seven in professional status, 10 in interaction and seven in the organizational policies subscale (Ahmad & Oranye, 2010; Stamps & Piedmonte, 1986). The reliability index of the IWS has been reported in previous studies (Ahmad & Oranye, 2010; Medley & Larochelle, 1995; Wade et al., 2008) and in the Manual (Stamps & Piedmonte, 1986). This study, conducted a systematic search of the literature related to nurses' job satisfaction and research in four major databases of PubMed, CINAHL, PsycINFO and SCOPUS from 1986 ‐ May 2015, to find studies that have relevance to this study and to ascertain if Rasch model has been applied to the IWS. The following inclusion and exclusion criteria were applied: (1). Papers published in English language; (2). Publications with a study sample that included nurses; (3). Job satisfaction was measured using the IWS; (4). Reliability and validity of the IWS were reported for the study sample; (5). Papers that applied Rasch analysis model to measures of job satisfaction. The search resulted in 100 papers, which were further screened for relevance. Finally, 53 of the papers and four other papers on Rasch model were included in this study.

### 2.3 Analysis

The Rasch analysis model was applied using the Rumm 2030 software. Data from the two countries were stacked for comparative analysis purpose. The Rumm 2030 software performs an item by item analysis, providing the capability to examine each item at different levels, including individual, country and other group levels, such as age, work status etcetera. The data stacking enables a simultaneous analysis of variables across the multiple levels. An analysis of the fit statistics was used to determine if IWS scale fits the Rasch model expectations. The statistics were examined to determine whether the criteria of unidimensionality, differential item functioning (DIF) and person separation index (PSI) were satisfied by the IWS scale (Brentari & Golia, 2008).

## 3 Results

### 3.1 Descriptive analysis

Of the 554 subjects in the data, 70% were from Malaysia and 30% were British. The majority of the subjects were female (96.4%), which is a reflection of the gender composition of the nursing profession in many environments. The majority were married (62.5%), while 34.5% were single and the others were divorced, widowed or unknown. A smaller proportion had a university degree (11%), but the most common level of education was Diploma (65.9%) and a certificate in nursing (23.1%). Most of the nurses worked as full time staff (90.4%).

**Figure 1:**
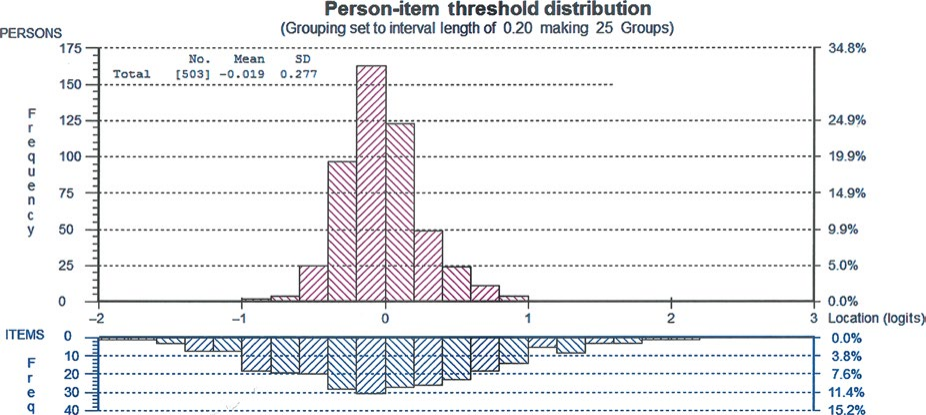
Person‐item threshold distribution

In Rasch analysis, one way to measure the adequacy of a tool is the targeting of the traits of interest in the population. In Fig. 1, the spread of the items in the scale vis‐à‐vis the persons location shows that the tool has a good targeting of the person characteristics in the sample. The persons mean location of −0.018 is approximately equal to the items mean of 0.00. However, the negative persons mean value suggests the possibility of very few participants whose scores were lower than the theoretical expected average level of job satisfaction. This could also suggests a slightly lower level of job satisfaction among the population.

### 3.2 Reliability indices

The Rasch model provides two estimates that confirm the reliability of a tool and the precision of the estimate of each person trait in the sample. The person separation index (PSI)=0.8578 was approximately equal to the Cronbach α coefficient=0.851, both of which indicate a very good reliability and internal consistency of the IWS (Table 1).

**Table 1:** Test of reliability

Scales	With extremes		Without extremes		*N*
	PSI	Conbach α	PSI	Conbach α	
All 44 items	0.8578	0.851	0.8578	0.851	503
Professional status	0.6441	0.5567	0.6101	0.5418	540
Task requirement	0.5824	0.5611	0.5747	0.5611	550
Pay	0.4814	0.4819	0.4275	0.4612	540
Interaction	0.7811	0.7429	0.7811	0.7429	544
Organizational policies	0.591	0.5575	0.5697	0.5502	545
Autonomy	0.7258	0.6853	0.7115	0.6791	546

PSI, Person Separation Index.

### 3.3 Fit analysis

Table 2 shows fit statistics for the item‐person interaction. The Rasch analysis indicates an excellent power of analysis of fit for the data, which means that the analysis was strong enough to detect any differences where there was one. The standard deviation of the fit residuals for the items at the subscale levels and the total scale were high, suggesting poor fit to the Rasch model. Generally, these suggest the presence of some mis‐fitting items and individuals in the data set whose response patterns deviated substantially from the expectation of the Rasch model (Tennant & Conaghan, 2007). In all the six subscales, the residual standard deviation for the items were higher than the residual standard deviations for persons. The persons residual standard deviations for the Professional status, Task requirement and Pay subscales are below 1.4, suggesting that it is very unlikely there were persons whose responses deviated significantly from the Rasch model expectation in those subscales. The significant Chi Square, *p* < .0001, equally indicates a misfit to the Rasch model. Cummings, Hayduk, and Estabrooks (2006) have argued that the lack of model fit in a measurement tool is an indication of a validity problem. The Rasch model identified 51 extreme cases, which were subsequently dropped from the analysis. The removal of these extreme cases did not alter the power of analysis fit and the Chi Square fit statistics remained significant. So, the misfiting was not caused by the extreme values. The RMSEA was calculated to further evaluate the model fit. The result shows that the Pay subscale has a poor fit to the Rasch model, while the two subscales of Interaction and Organizational Policies closely approximate the Rasch Model. For the other three subscales, there is a reasonable error of approximation to the model fit (Browne & Cudeck, 1993).

**Table 2:** Summary test‐of‐fit on item‐person interaction

Scales	Items		Persons		RMSEA	χ^2^	*p* value
	LocationMean (*SD*)	ResidualMean (*SD*)	LocationMean (*SD*)	ResidualMean (*SD*)			
Total scale	0.00 (0.39)	0.63 (1.65)	−0.02 (0.26)	−0.42 (2.34)	0.069	1189.42	<.0001
Professional status	0.00 (0.31)	0.76 (1.62)	0.35 (0.5)	−0.29 (1.13)	0.055	146.33	<.0001
Task requirement	0.00 (0.48)	−0.03 (1.91)	−0.31 (0.51)	−0.4 (1.08)	0.053	121.02	<.0001
Pay	0.00 (0.39)	0.59 (3.36)	−0.42 (0.43)	−0.36 (1.15)	0.115	390.5	<.0001
Interaction	0.00 (0.37)	0.78 (1.61)	0.23 (0.53)	−0.37 (1.42)	0.036	135.06	<.0001
Organizational policies	0.00 (0.19)	0.83 (1.57)	−0.28 (0.43)	−0.46 (1.48)	0.045	102.21	<.0001
Autonomy	0.00 (0.23)	0.31 (2.25)	0.11 (0.53)	−0.56 (1.58)	0.058	180.5	<.0001

*SD*, standard deviation; RMSEA, root mean square error of approximation.

#### 3.3.1 Unidimensionality test

Another important statistics considered in this study is the unidimentionality test, using the paired *t* test statistics. The paired *t* test = −2.8, shows that 187 of the sample estimates were significantly different at *p* < .05 and 110 were significantly different, at *p* < .01. The *t* test statistic gave a significant value much higher than the 5% required for Rasch unidimensionality. This analysis supports the multidimensionality of the IWS scale, which was originally designed to measure six dimensions of pay, autonomy, task requirement, organizational requirement, job status and interaction.

The individual item fit residuals were examined to identify those items that may be causing the model miss fit. The result from the subscales shows that items 7, 10, 14, 18, 32, 36 and 43 had extreme fit residual values. The fit residuals for items 7 and 32 were consistently high at subscale and combined scales levels. Also, the Table 3 shows that a total of 12 items had significant Bonferroni Adjusted χ^2^ probability <0.00125, indicating that these items were miss fitting of the Rasch model.

**Table 3:** Analysis of miss fitting items based on subscales

Subscales	Item	Location	*SE*	FitR	*df*	χ^2^	*df*	*p* values
Task requirement	22	−0.67	0.04	2.43	454.5	46.87	8	<.0001*
	36	0.50	0.04	−2.57*	454.5	18.6	8	.0172
Pay	01	−0.2	0.03	−0.21	443. 7	27.68	8	.0005*
	14	0.08	0.03	−2.57*	443. 7	69.24	8	.0000*
	21	0.05	0.03	0.01	443. 7	28.6	8	.0004*
	32	−0.64	0.03	6.75*	443. 7	215.09	8	<.0001*
	44	0.53	0.04	−1.997	443. 7	37.3	8	<.0001*
Interaction	03	−0.05	0.03	3.83	485.7	11.85	8	.1582
	10	0.15	0.03	3.26*	485.7	14.22	8	.0762
Autonomy	07	−0.19	0.03	2.26*	473	26.61	8	.0008*
	31	0.00	0.03	−2.5	473	25.71	8	.0012*
	43	0.02	0.03	3.43*	473	23.33	8	.003
Organizational policy	18	−0.1	0.03	2.62*	462.4	11.00	7	.1384
	25	0.00	0.03	−1.19	462.4	25.87	7	.0005*
	33	−0.01	0.03	2.63	462.4	20.13	7	.0051
Professional status	02	0.47	0.03	3.66	456.4	34.38	8	<.0001*
	09	0.04	0.03	0.60	456.4	29.29	8	.0003*
	15	−0.47	0.04	−0.9	456.4	30.42	8	.0002*

Note * denotes significant p values. *SE,* standard error; FitR, Fit Residual.

#### 3.3.2 Analysis of Differential Item Functioning (DIF)

The DIF is a test of item bias or how each item in the scale functions for each individual, irrespective of ‘ability level'. In this study, the DIF was examined with respect to age, gender, years of experience, work status (full time or part time) and country. A primary factor of interest is the country, whether participants were British or Malaysian nurses, which by extension implies socioeconomic and cultural differences.

Table 4 shows items with significant DIF for the six person factors. The difference between participants was considered significant, if the *F*‐statistics has an adjusted Bonferroni probability <.001667. A total of 18 items had significant DIF at country level, 10 of which have significant or high fit residuals. It is known that the presence of DIF can cause a misfit to the model. One item, 42 had significant DIF for sex, *p* = .0014. A few items showed evidence of significant DIF for age, work status, years of experience, education and marital status. The large number of items with significant DIF for country raises questions about the cross‐cultural validity of the IWS tool.

**Table 4:** Analysis of DIF by subscale

Subscales	Items	Country		Age*p* value	Work status*p* value	Experience*p* value	Education*p* value	Gender*p* value	Marital status*p* value
		*F*	*p* value						
Professional status	2	11.0799	.0009						
	15			<.0001		<.0001			
	27	84.3628	<.0001				.0011		
	38	20.0570	<.0001						
	41	27.485	<.0001						
Task requirement	4			.0006		.0009			
	22	31.1457	<.0001	<.0001		.0001	.0002		
Pay	1	19.4680	<.0001						
	32	76.4063	<.0001						.0012
	44	52.5904	<.0001						.0015
Interaction	6	13.2544	.0003						
	19	21.8351	<.0001						
	35	21.5627	<.0001						
Organizational policies	18	27.6627	<.0001						
	33	23.9983	<.0001						
	40	26.5460	<.0001						
	42	61.7807	<.0001					.0014	
Autonomy	7	50.2147	<.0001		.0002				
	13			<.0001		<.0001	.0006		
	17					.0016			
	26						.0003		
	31	13.9779	.0002						
	43	82.4976	<.0001						

Note. The p values reported in the table were below adjusted bonferroni probability 0.001667. *Lowest adjusted bonferroni probability <.001667 for all the subscales.

One‐way ANOVA was performed to determine the significance of the variation in the IWS items with regard to the person factors. The result in Table 5 shows significant variation at country and education levels, adjusted Bonferroni probability <.000379. The high significant variation for country, *p* < .0001 supports the result from Table 4 that the IWS functions very differently for nurses in Malaysia than those in the UK.

**Table 5:** ANOVA for DIF in participants

Factor	*F*	*df*_B_	*df*_W_	*p* value
Country	98.93	1	501	.0000
Age	4.84	3	499	.0025
Education	8.97	2	500	.0002
Experience	4.82	3	499	.0026
Sex	4.76	1	501	.0295
Marital status	3.22	2	500	.0408
Work status	1.55	1	501	.2132

Note. p values for the subscales are significant if below 0.000379. *Adjusted Bonferroni probability <.000379 for all the 44 items.

**Figure 2:**
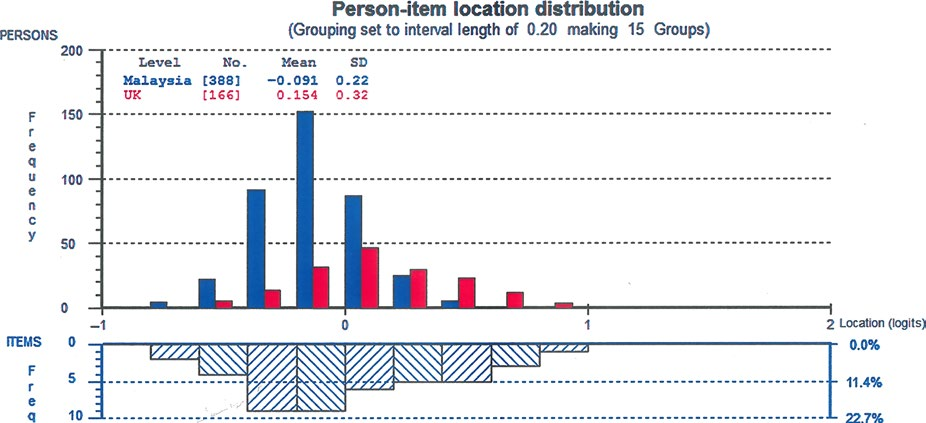
Person‐item distribution by country

The person‐item distribution (Fig. 2) shows that Malaysian nurses had lower levels of job satisfaction, with a group mean = −0.091, compared with the group mean = 0.158 for UK nurses. The wide spread of the items could mean that some of the items may not be relevant for understanding the constructs, given that there were many measures on the two extremes.

## 4 Discussion

The PSI and Cronbach's α for the Index of Work Satisfaction in this study are consistent with previous studies, which reported good reliability, ranging from 0.54–0.92 (Bjork et al., 2007; Curtis & Glacken, 2014; Manojlovich & Laschinger, 2007; Oermann, 1995; Stamps & Piedmonte, 1986). There is a strong evidence, both from this and previous studies that support the reliability of IWS for assessing job satisfaction among nurses. However, it is possible that the variation in the reliability reported across studies is an indication that the meanings or values of some of the items may not always be consistent across populations. For instance, Karanikola and Papathanassoglou (2015) found that two items in the IWS scale were not consistent with other items and as such affected the internal consistency of the tool. Essentially, the reliability of a measurement tool focuses on the consistency of the measurement in measuring what it is intended to measure. However, what is measured, especially in the social world, is often inequivalent across social environments, because the meanings and values vary from one place to another. Therefore, it is important that attention is paid not just to the consistency of a scale, but the meanings and values of the concept or construct being measured, across cultures.

The high standard deviations of the fit residual for the items (range: 1.57–3.36) points to the possibility of mis‐fitting items, while the fit residual for the persons (range: 1.08–1.58) shows the less likelihood of individuals whose response patterns deviated substantially from the expectation of Rasch model, compared with the items (Tennant & Conaghan, 2007). The Chi Square statistics and RMSEA were used to determine if the person‐item interaction in the IWS provides a good fit to the Rasch model. While the significant Chi Square, *p* < .0001, indicates a deviation from the Rasch model, the RMSEA suggests a mixed bag, with some subscales presenting a better fit than others. Apart from the Pay subscale which has a poor fit to the Rasch model, the two subscales of Interaction and Organizational Policies closely approximate the Rasch Model. The other three subscales of Autonomy, Professional Status and Task Requirement demonstrated reasonable errors of approximation to the model fit (Browne & Cudeck, 1993), which may not necessarily imply a complete misfit to the Rasch model. Cummings et al. (2006) have pointed out that the lack of model fit in a measurement tool is an indication of validity problem. There are several reasons why a measurement tool or data may have a model misfit. The lack of Fit to the Rasch Model in this study could be due to cultural differences between the two countries, the size of the sample or because the IWS is a multidimensional scale. It is also known that the presence of DIF can cause a misfit to the Rasch model. All of these factors are true in this study analysis. Essentially, our primary interest in this analysis was to determine whether the IWS tool functions differently for different groups (DIF), whether the groups are at country level, gender, work status etc. Some of the items identified in this study would require further analysis, to determine why they have high or significant fit residual.

The IWS items align very well with the persons measure, but overall, the spread of the items were wider on both tails of the graph (Fig. 1) than the person traits being measured. The spread seems to suggest that some of the measures were either above or below the respondents' ‘ability' level. In the context of the measurement of job satisfaction, the items at the extreme were possibly measuring traits that may not be directly relevant to understanding participants' job satisfaction. Again, it is important to note that what makes for job satisfaction would very likely vary in time, place and people. A detailed individual item‐response analysis will be required to identify those items in the tool that are probably irrelevant or contributing very little to the measurement of the construct of job satisfaction or the underlying concepts of pay, professional status, interaction, task requirements and organizational policies.

The Rasch model expects a good measurement tool to be invariant across the sample and traits being measured. In other words, each item on a measurement scale is expected to measure the attribute of interest between different participants without any bias. Linacre and Wright (1987) have emphasized the importance of identifying and quantifying differential item functioning for contrasting groups and to clearly understand the differences between groups. For a measurement tool, such as the IWS that is designed to be used in different environments, it is important to understand how the different items in the tool function for different participants and groups. The presence of a substantial number of items with significant DIF, *p* < .0017 for participants from Malaysia and UK in this study is an important measurement issue that researchers who use the IWS scale need to pay attention to. A significant DIF could be an evidence of measurement bias, or may result from the nature of the constructs being measured. Such a bias will have important implications for the cross‐cultural validity of how job satisfaction is being measured by IWS. Also, it should be noted that the meanings of the concepts of payment, professional status, task requirements, professional interactions, organizational policies and autonomy could differ for the nurses in these two countries. The meanings of the item questions in each of the six components and the values of what is being measured may not be equivalent across cultures and countries. This significant DIF for the countries raises another important question on the use of IWS for measuring and comparing nurses' job satisfaction across cultural groups and whether the findings from different countries are actually comparable or generalizable across countries, or even among cultural groups in the same country or workplace. Belvedere and de Morton (2010) have pointed out that the presence of DIF calls to question the validity and generalizability of a measurement result.

The negative mean of −0.091 indicates that on the average, job dissatisfaction was lower among nurses in Malaysia than those in the UK. Ahmad and Oranye (2010) have reported a significant difference in job satisfaction between the English and Malaysian nurses and pointed out that the factors that determine job satisfaction was different for both groups. Several studies (Adwan, 2014; Alvarez & Fitzpatrick, 2007; Andrews, Stewart, Morgan, & D'Arcy, 2012; Ea et al., 2008) have reported total job satisfaction based on the IWS tool. Adwan (2014) reported high scores in most of the IWS subscales among paediatric patient care nurses, while Alvarez and Fitzpatrick (2007) reported (67%) moderate job satisfaction and (33%) low job satisfaction at the unit levels. The calculation of total scores on job satisfaction was made on the assumption that the scores on the subscales are additive. However, given the multidimensionality of the IWS scale and the differences in the meanings of what is being measured, it is questionable that total scores are realistically comparable. The study by Ahmad and Oranye (2010) points to the fact that what determines job satisfaction can vary between groups and countries. For instance, while the pay was the significant determinant of job satisfaction among the English nurses, ‘interaction—the opportunities presented for both formal and informal contacts during working hours' (Ahmad & Oranye, 2010, p. 589), was the primary determinant of job satisfaction among Malaysian nurses. These differences in perceived job satisfaction may be a function of DIF as evident in this study, than other workplace or condition of work factors.

### 4.1 Limitations

There were 51 extreme cases in this study sample; however, their removal did not significantly change the result. The findings from this single study may not be sufficient to draw definitive conclusions on the miss fit of IWS to the Rasch model. Further studies across countries and work environments that apply Rasch model and a review of local dependency and item difficulty levels is needed.

## 5 Conclusion

The IWS is a very reliable tool, especially at the composite level, as indicated by this study and several others. The IWS has been used in several nursing studies, but its cross‐cultural validity has not been well evaluated based on item‐response theory and using Rasch model statistics of DIF. Caution should be exercised in comparing results of IWS across cultural groups, in view of the evidence on possible DIF for culturally diverse societies. It is important that further studies are conducted to test for DIF across cultural groups. Given that the IWS is a multidimensional tool, it may not be realistic to sum up the scores from the different dimensions as an index for comparison between significantly different groups, since the issues they measure may vary over time and place. Equally, it may not be meaningful to compare the total score on job satisfaction between two different groups, since the meaning of job satisfaction or any of the components may differ significantly between groups.

## Funding

There is no funding for this study.

## Conflict of interest

The authors declare that there is no conflict of interest in this study.

## Author contributions

All authors have agreed on the final version and meet at least one of the following criteria [recommended by the ICMJE (http://www.icmje.org/recommendations/)]:

- substantial contributions to conception and design, acquisition of data, or analysis and interpretation of data;
- drafting the article or revising it critically for important intellectual content.
